# Brain histamine and behavioral neuroscience

**DOI:** 10.18632/oncotarget.15365

**Published:** 2017-02-15

**Authors:** Andrea Santangelo, Maria Beatrice Passani, Maurizio Casarrubea

**Affiliations:** Department of Neuroscience, Psychology, Drug Research and Child Health, Psychiatric Unit, University of Florence, Florence, Italy

**Keywords:** histamine, behavior, T-pattern, behavioral sequence, Neuroscience

The human histaminergic system contains approximately 64.000 neurons located in a posterior region of the hypothalamus, the tuberomamillary nucleus. Projections of such a relatively small number of neurons are surprisingly widespread throughout the central nervous system. Histaminergic neurons are highly active during wakefulness and contribute to the physiology of arousal state, exploration, pain modulation, nutrition, thermoregulation and brain energy metabolism, learning and memory functioning [1]. The possibility to assess the contribution of brain histamine (HA) to specific behavioral changes is a complex task, taking into consideration the broad modulatory activity exerted by this amine at different levels of cortical and subcortical circuitry. An explanation of the diversity of functions of the histaminergic system may be attributed to the heterogeneous nature of HA neurons that are organized into functionally distinct circuits, impinging on different brain regions, and display selective control mechanisms [2].

From a quantitative perspective, data from genetic models indicate that lack of HA is associated with reduced motor activity reasonably due to impaired arousal state and motivation [3]. Recent findings have unveiled an additional relevant contribution of brain HA on qualitative features of the displayed motor behavior both in humans and animals [4]. Ercan-Sencicek and colleagues [5] have elaborated a histaminergic hypothesis of tic phenomenology on the basis of the identification of a rare autosomal dominant form of Tourette’s syndrome highly correlated with a nonsense mutation, Hdc W317X, on the gene for histidine decarboxylase (HDC) the key-enzyme responsible for HA synthesis. HA and the H3-receptor have a strong influence on dopaminergic activity in the Basal Ganglia suggesting an unexpected role of HA in the pathophysiology of movement disorders, not only confined to tic symptomatology. The peculiar distribution of the H3 receptor, mainly confined to the brain, calls for a better understanding of the role of this amine in brain circuitry [6]. The H3 receptor likely represents a selective pharmacological target to modulate brain HA. Albeit a direct involvement of the histaminergic system has been suggested, to our knowledge, only in the Tourette’s syndrome, markers of an altered HA activity have been described in several neuropsychiatric conditions characterized by stereotypical or abnormal motor behavior and thoughts, such as Schizophrenia, Parkinson’s disease, Huntington’s disease or Addiction [3][6].

In view of these results, we have recently described the effect of an acute brain HA depletion on the display of motor sequences of naïve CD1 mice in the open-field [7]. Our approach does not require any previous conditioning and the analysis is carried out following a high-res video recording of the ongoing behavior. HA depletion was obtained by intracerebroventricular administration of the HDC inhibitor alpha-fluoromethylhistidine and motor sequences were detected and analyzed using a multivariate technique known as temporal pattern (t-pattern) analysis. Basically, a t-pattern represents a sequence describing repetitive aspects of a studied behavior [8] and can be formally presented using the following expressions:

$$X_{1}\approx dt_{1}\;X_{2}\approx dt_{2}\;X_{3}\ldots X_{i}\approx dt_{i}\;X_{i+1}$$

where, X_1_ … X_2_ … X_3_ indicate the components of a given sequence of events and ≈dt terms indicate the temporal interval between these components (with ≈dt ≥ 0). Hence, for instance, X_i_ ≈dt_i_ X_i+1_ indicates a t-pattern where the component X_i_ is followed by the component X_i+1_ after the dt_i_ interval.

**Figure 1:**
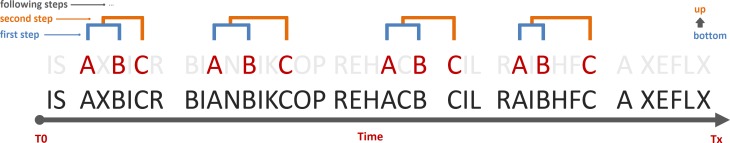
Example of a three-event t-pattern occurring four times during an observation period (T0-Tx) encompassing a 40 hypothetical events (letters) The ((A B) C) sequence becomes evident if all the remaining events are left out (grey letters). “First”, “second” and “following steps” = search runs carried out by the software on the basis of a bottom-up criterion.

T-pattern analysis is performed by means of a specific software known as Theme^®^ (Patternvision LTD, Iceland; Noldus IT, The Netherlands). This computer program, on the basis of an advanced algorithm, searches for statistically significant constraints among events in observational data, by taking into account occurrences, order, and timing of the events. For example, given a hypothetical period of observation encompassing a number of behavioral events (e.g. A, B, C, D, …) performed by a freely moving animal (see Figure 1), the algorithm compares the distributions of each pair of events (e.g. “A” and “B”) assessing the existence of a time interval so that “A” is followed by “B” within that interval more often than chance expectation. If such a circumstance is confirmed, “A” and “B” are considered a t-pattern of first level (A B). In a second step, following a bottom-up search process, this pattern is considered the starting point for higher-order t-patterns, e.g. ((A B) C) and so on up to any level. Fig. 1 illustrates a hypothetical t-pattern of three events, occurring four times within an observation period encompassing several behavioral events. More details concerning theories, concepts and techniques behind t-pattern analysis can be found in our recent book [8]. By using such a multivariate technique, main results of our study [7] can be summarized as follows:

- Brain HA has important modulatory activity in terms of behavioral patterning/sequencing;
- Acute HA depletion is associated with increased number and complexity of motor sequences displayed in the open field;
- This enhancement appears to involve mainly exploratory, rather than grooming activity;
- The dopamine D2/D3 receptor antagonist sulpiride, at non sedative doses, provides a partial reversal of the effects observed in HA depleted mice, thus suggesting a relevant interplay between dopamine and histamine.

According to these results we suggest that the consistent increase both in complexity and number of exploratory sequences observed in the HA depleted animals, although not apparently associated with the presence of any actual abnormal motor behavior compared to controls, may represent the behavioral phenotype underlying the pathophysiology of tic-phenomenology and, possibly, other above-mentioned neuropsychiatric disorders. Further studies are in progress in our laboratories in this direction.
